# Stereotactic ablative body radiation therapy for isolated pulmonary metastases from pancreatic cancer after metastectomy with positive margins: a case report

**DOI:** 10.1186/s13256-023-03977-z

**Published:** 2023-06-14

**Authors:** Anoud Alnsour, Hien Le, Adam Byrne, Nick Rodgers, Daniel Roos

**Affiliations:** 1 Department of Radiation Oncology, Royal Adelaide Hospital, Port Rd, Adelaide, SA 5000 Australia; 2 Department of Radiation Oncology, King Hussein Cancer Center, Amman, Jordan; 3 Clinpath Laboratories, 21 James Congdon Drive, Mile End, Adelaide, SA 5031 Australia; 4 University of South Australia, Adelaide, Australia; 5 Department of Medicine, University of Adelaide, Adelaide, Australia

**Keywords:** Pancreatic cancer, Pulmonary oligometastases, Microscopically positive margin, Lung SABR, Case report

## Abstract

**Background:**

Isolated pulmonary oligometastases as the first site of dissemination after initial resection of pancreatic ductal adenocarcinoma (PC) is a rare event, and the treatment in this subgroup is challenging. Recurrence in the lung after initial primary tumour resection is associated with the most long-term survivors of patients with metastatic PC. Stereotactic ablative body radiation therapy (SABR) or metastectomy for pulmonary oligometastases from PC is becoming more common. However, patients with close or positive margins after metastectomy for isolated pulmonary metastatic PC are at high risk for recurrence. This requires a treatment capable of achieving high rates of local control and improved quality of life by delaying the need for systemic chemotherapy. In other settings, SABR has been shown to achieve these goals, allowing safe dose escalation with excellent conformity and short duration of treatment.

**Case presentation:**

We report the case of a 48-year old Caucasian man with a history of locally advanced PC initially treated with neoadjuvant chemotherapy followed by Whipple’s resection in August 2016. After a disease-free interval of 3 years, he developed three isolated pulmonary metastases which were treated with local resection. In the setting of microscopically positive resection margins (R1), adjuvant lung SABR was delivered to all three sites. His treated lung disease remained radiologically stable for up to twenty months after SABR. Treatment was well tolerated. In January 2021, he developed a malignant pre-tracheal node which was treated with conventionally fractionated radiotherapy and remained controlled for the duration of follow-up. A year later, he developed widespread metastatic disease including pleura, bone and adrenal gland, together with presumed progression in one of the original lung lesions, receiving palliative radiotherapy for right chest wall pain. He was later found to have an intracranial metastasis and died in February 2022, 5½ years after initial treatment.

**Conclusion:**

We present the case of a patient treated with SABR after R1 resection of 3 isolated pulmonary metastases from PC, with no treatment toxicities and durable local control. For well-selected patients in this setting, adjuvant lung SABR may be a safe and effective treatment option.

## Background

The majority of pancreatic cancers (PC) present with metastases and carry a high mortality rate. Despite decades of research aiming to improve the prognosis of advanced PC, the 5-year survival rate remains low [1]. However, recurrence in the lung after initial primary tumour resection is associated with favourable long-term survival compared with liver metastases and the majority of other metastatic sites [2]. The indications for metastectomy or Stereotactic Ablative Body Radiation Therapy (SABR) for pulmonary oligometastases in PC are not as well defined as for colorectal cancer with isolated pulmonary metastatic disease [3]. However, recent advances in effective therapy for PC have led to individual patients where metastectomy or SABR may potentially be indicated. Furthermore, patients with close or positive margins after metastectomy for isolated pulmonary metastatic PC are at high risk for recurrence, making their treatment challenging and requiring a treatment capable of achieving a high local control rate and better quality of life by delaying the need for systemic chemotherapy.

In other settings, SABR has been shown to achieve these goals, allowing safe dose escalation with excellent conformity and short duration of treatment. In this report we discuss a case of lung SABR for isolated pulmonary metastases after local surgical resection with microscopically positive margins and evaluate the efficacy and safety of this treatment option.

## Case presentation

A 48-year old Caucasian man with locally advanced PC was treated with five cycles of neoadjuvant FOLFIRINOX chemotherapy followed by pancreaticoduodenectomy (Whipple's procedure) in August 2016. Histopathology confirmed moderate to poorly differentiated adenocarcinoma with evidence of perineural but no lympho-vascular infiltration. Margins were clear, and the UICC staging was *ypT1N1M0* (AJCC 7th Edition.) He then had one cycle of adjuvant FOLFIRINOX (minus oxaliplatin due to grade 2–3 peripheral neuropathy from prior chemotherapy), but suffered significant side effects, precluding further adjuvant chemotherapy.

**Fig. 1:**
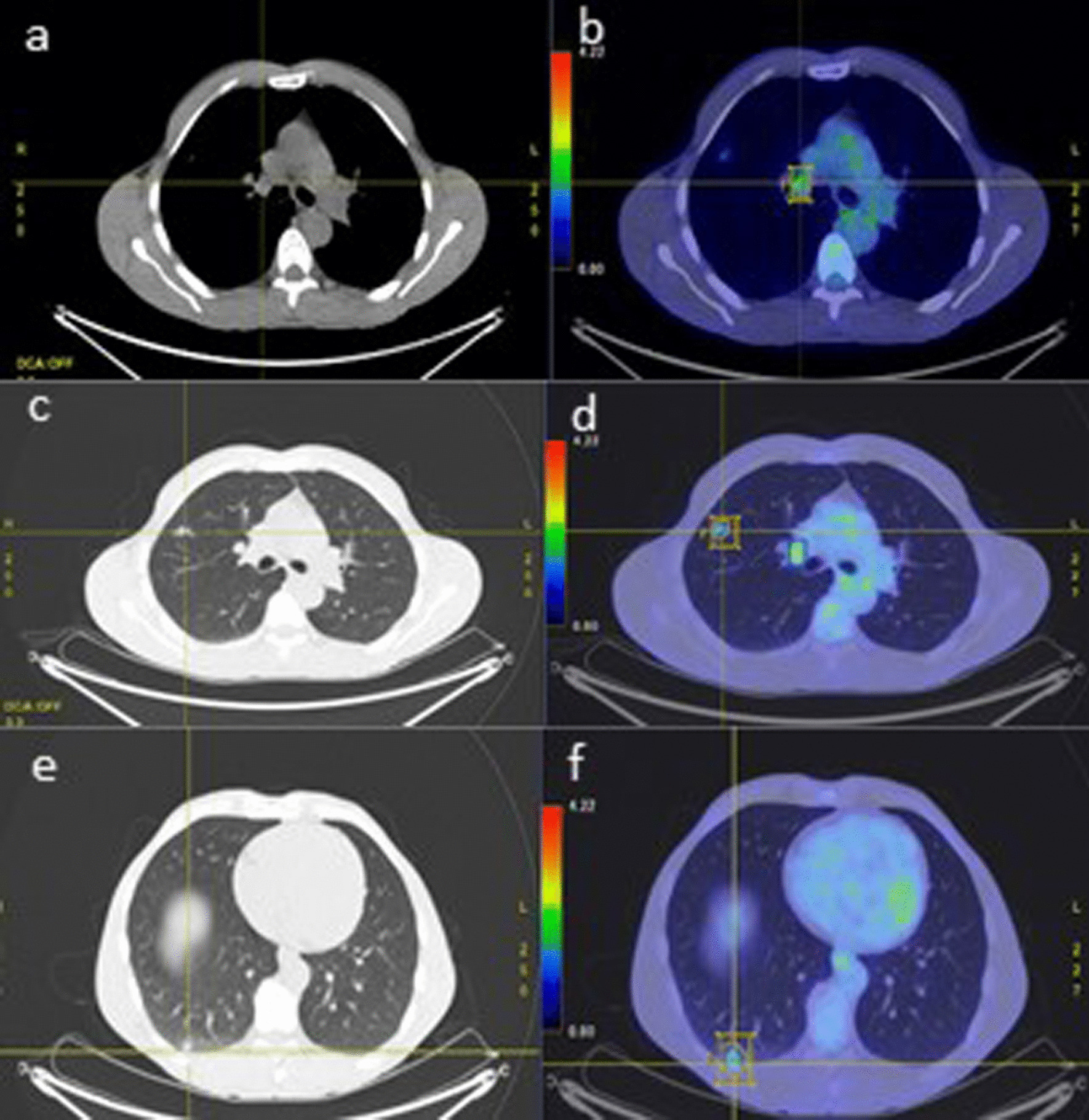
Axial positron emission tomography-computed tomography October 2019. **a**, **b** Right hilar node with equivocal fluorodeoxyglucose uptake on PET. **c**, **d** Fluorodeoxyglucose avid right upper lobe nodule. **e**, **f** Fluorodeoxyglucose avid mass in the right lower lobe

Follow-up clinical examination, blood tests and computed tomography (CT) were performed every 3 months. Tumour markers carbohydrate antigen (CA) 19-9 and carcinoembryonic antigen (CEA) remained within the normal range. In February 2019, a small (5 mm) lung nodule was noted with subtle increase in size over 1 year. Eventually, in October 2019, a PET scan (Fig. 1) demonstrated low grade but suspicious fluorodeoxyglucose (FDG) uptake in right lung upper (SUV max 1.7) and lower lobe (SUV max 1.8) nodules and an equivocal right hilar node (SUV max 2.4), with no evidence of loco-regional recurrence or other distant metastases. The patient remained asymptomatic during this period.

The right hilar node was biopsied, confirming metastatic adenocarcinoma consistent with a gastrointestinal tract primary. He underwent video-assisted thoracoscopic right hilar node resection, wedge resections of the right upper and lower lobe lesions, and excision of a right para-tracheal node in February 2020, with uneventful recovery.

**Fig. 2:**
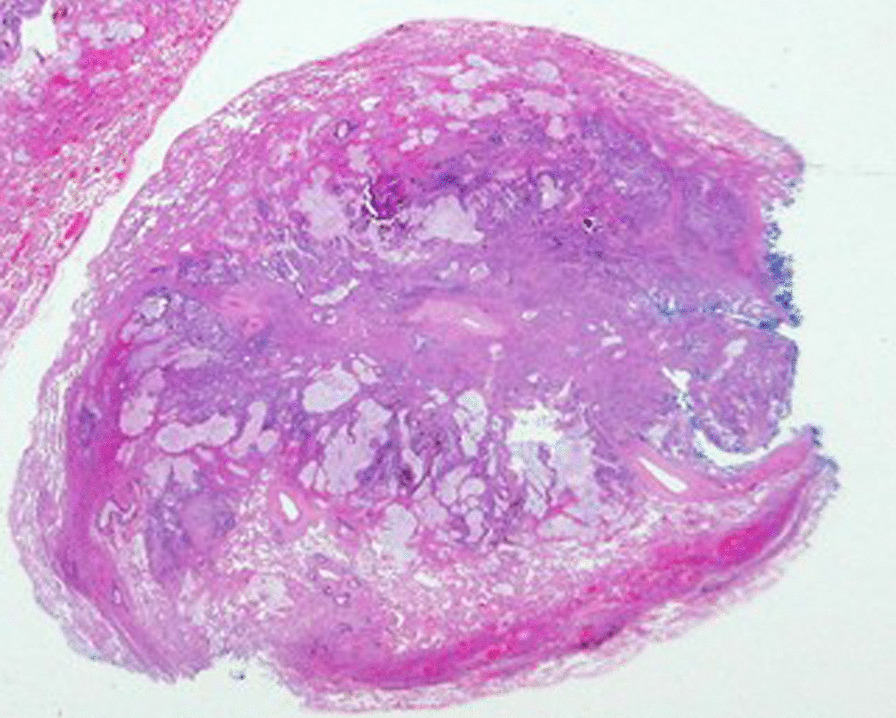
Extensive replacement of lung parenchyma centrally by metastatic adenocarcinoma with extensive extracellular mucin. Compressed normal lung parenchyma is evident at the perimeter

Histopathology was consistent with metastatic moderate to poorly differentiated adenocarcinoma in the right hilar node and in the two right lung parenchymal metastases, all three with positive margins. The hilar lesion also demonstrated extra-nodal extension (Fig. 2). The tumours were negative for both TTF1 and Napsin A, supporting the diagnosis of metastatic PC. The para-tracheal node was negative for malignancy.

He was referred to the Radiation Oncology clinic for consideration of SABR. The treating medical oncologist favoured radiotherapy over systemic therapy, given the low burden of disease as well as his documented chemotherapy related toxicities. He had excellent performance status (ECOG 0) and denied any respiratory symptoms. He lived at home with his wife and lived an active lifestyle. He was a non-drinker and a non-smoker. There was no other significant past medical history apart from depression and physical examination was unremarkable. His medications prior to diagnosis included tramadol 50 mg bd, diazepam 5 mg bd, mirtazapine 60 mg od and a daily adjusted dose of Creon. Recent pulmonary function tests were normal (FEV1 4.6, DLCO 76%).

His case was discussed at our departmental SABR meeting. Taking into consideration his relatively long disease-free interval, good performance status, and relatively young age, as well as the positive surgical margins and extra-capsular nodal extension of the right hilar node, SABR was advised to all three sites aiming to decrease the chance of local recurrence.

**Fig. 3:**
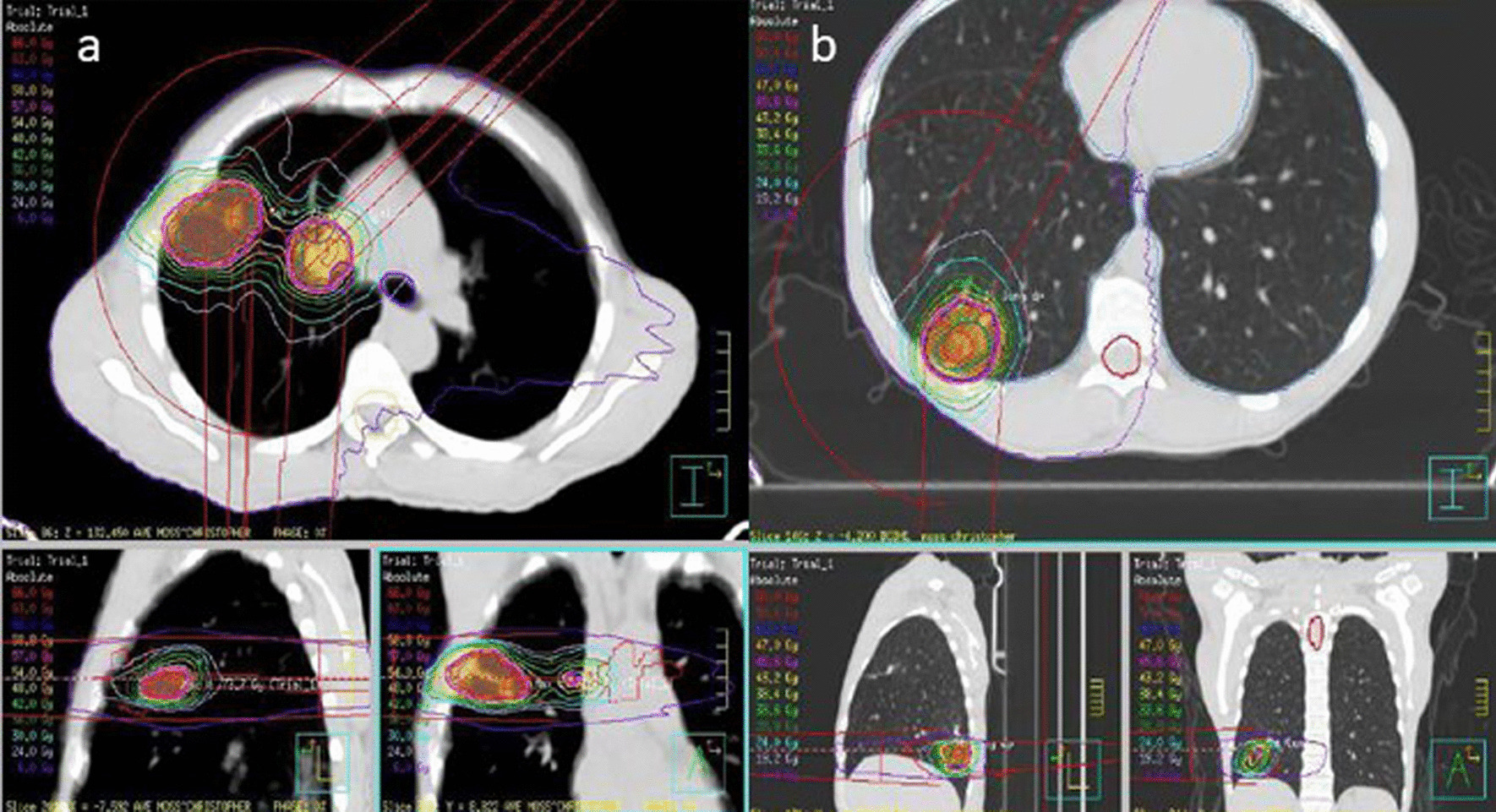
Computed tomography planning scan and isodose lines representing radiation dose distribution. **a** Planning target volumes 1 and planning target volumes 2 (hilar nodal bed and right upper lobe tumour bed, respectively). **b** Planning target volumes 3 (right lower lobe tumour bed)

The patient underwent treatment in April 2020. Simulation utilized a free breathing 4-Dimensional CT (4D-CT) technique. Internal target volumes were created based on the 4D-CT. The surgical bed was delineated with the aid of surgical clips and pre-operative diagnostic imaging which was fused to the 4D-CT. For planning target volumes (PTV) 1 and 2 (hilar nodal bed, 26.5 cc, and right upper lobe tumour bed, 34.2 cc, respectively), the dose prescription was 60 Gy in 15 fractions to the 91% isodose line for each; PTV3 for the lower lobe lesion (38.5 cc) was prescribed 48 Gy in 4 fractions to the 86% isodose line. Treatment was delivered over three weeks with sessions at least 48 h apart using a flattening filter-free linac based volumetric modulated arc radiotherapy (VMAT) technique (Fig. 3).

Daily on-board imaging was utilized with cone‐beam CT matching with 0 mm tolerance for couch shifts.

**Fig. 4:**
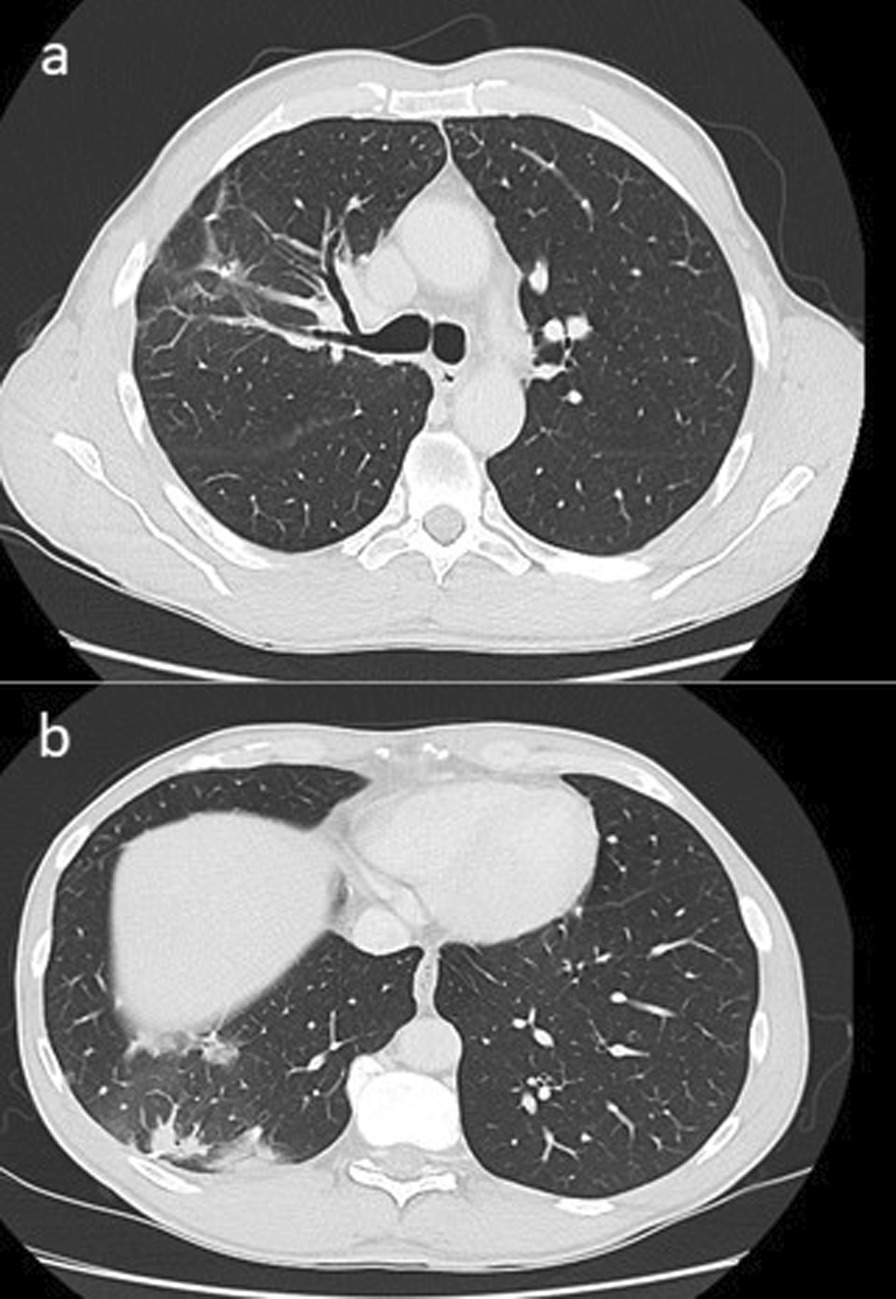
Axial slices of computed tomography chest 6 months post-SABR. Post-surgical and radiotherapy changes in the right lung with no evidence of progression. **a** Right upper lobe. **b** Right lower lobe

The patient tolerated radiation well, with no acute or late side effects. He was followed up at 3-monthly intervals, disease control defined by stable findings on CT scan in the absence of clinical or biochemical evidence of progression. CT scans 3 and 6 months post-SABR demonstrated no evidence of recurrence (Fig. 4) and CA 19-9 remained normal until the last reading of 2 U/mL (normal range 0–37 U/mL) which was taken on 14/9/2021.

**Fig. 5:**
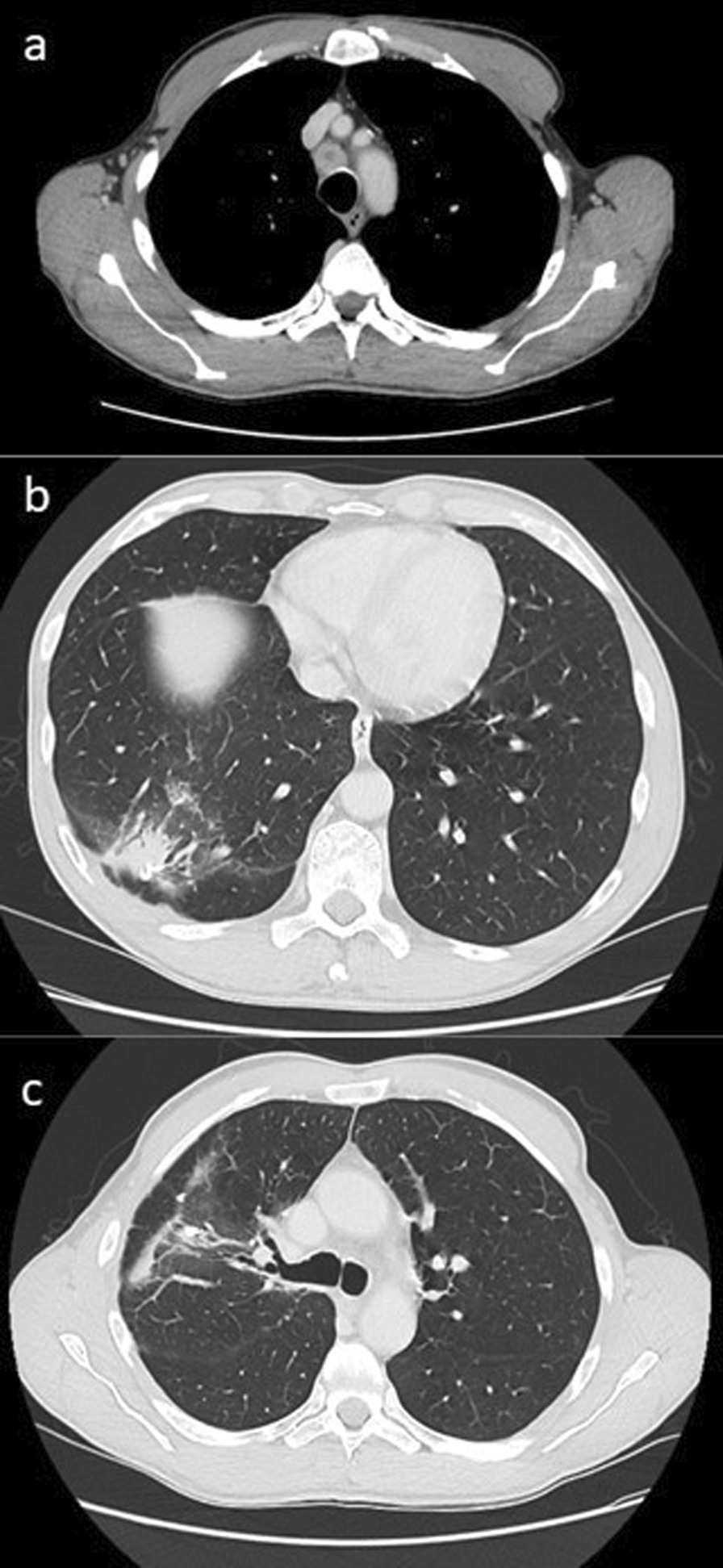
**a** Enlarging pre-tracheal lymph node with necrotic core. **b**, **c** Stable findings in the right lung

**Fig. 6:**
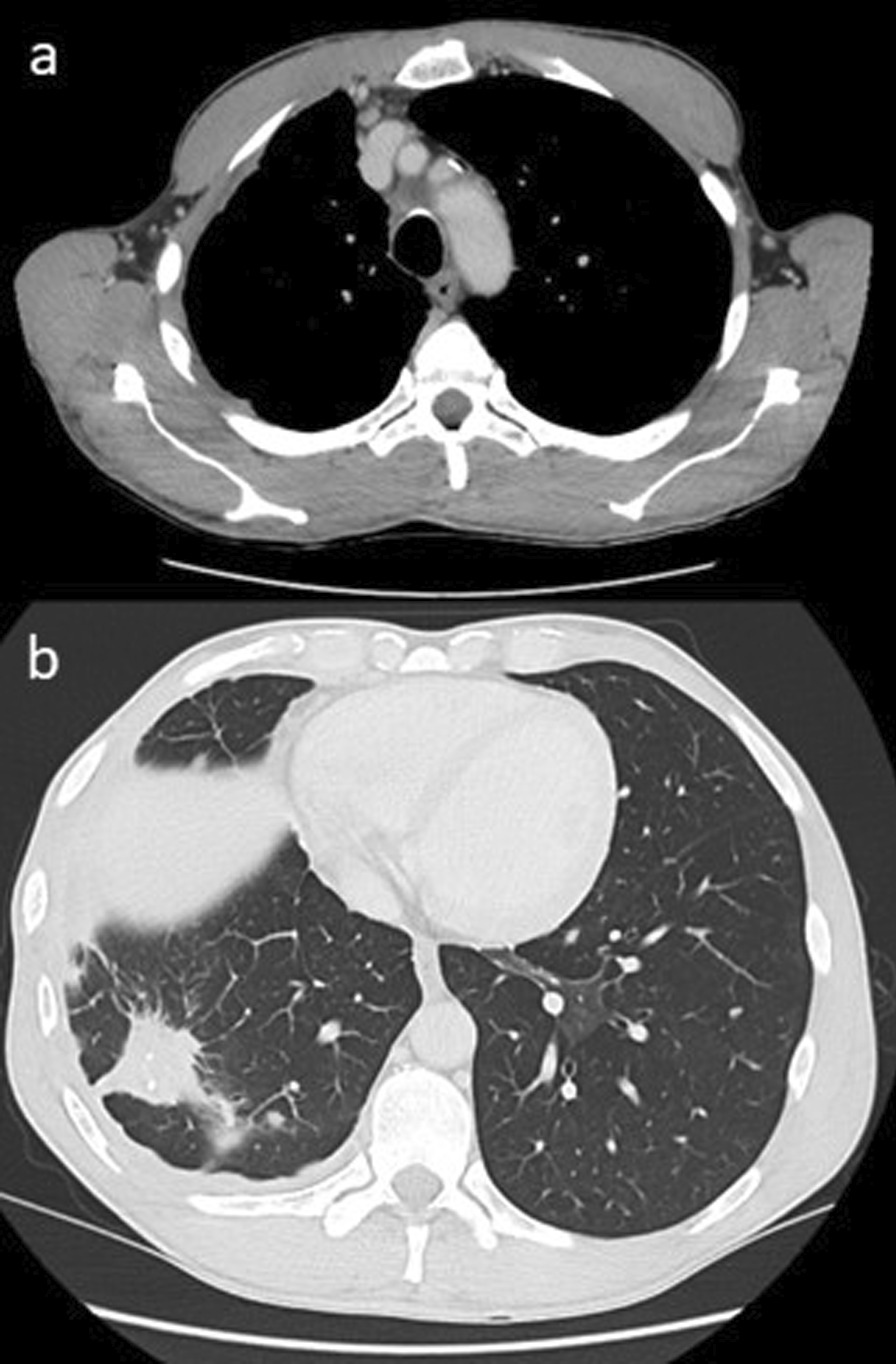
**a** The necrotic pre-tracheal node has responded to radiotherapy. **b** Soft tissue opacity in the treated region of the right lower lobe

The 9 month follow up CT in January 2021 was stable with respect to the areas of the right lung treated with SABR, but did show an enlarging pre-tracheal lymph node measuring 15 mm in the short axis (Fig. 5). This was confirmed to be consistent with metastatic PC via endobronchial ultrasound biopsy. He underwent high dose palliative RT to this isolated metastatic site, receiving 54 Gy in 30 fractions in March–April 2021. CT chest in June 2021 (Fig. 6) showed response in the treated node, now measuring 11 mm. A developing soft tissue opacity in the previously treated region of the right lower lobe was considered consistent with post-treatment effect/pseudoprogression and further surveillance imaging was favoured. Some nodularity of the right lung pleura was also noted at this time.

**Fig. 7:**
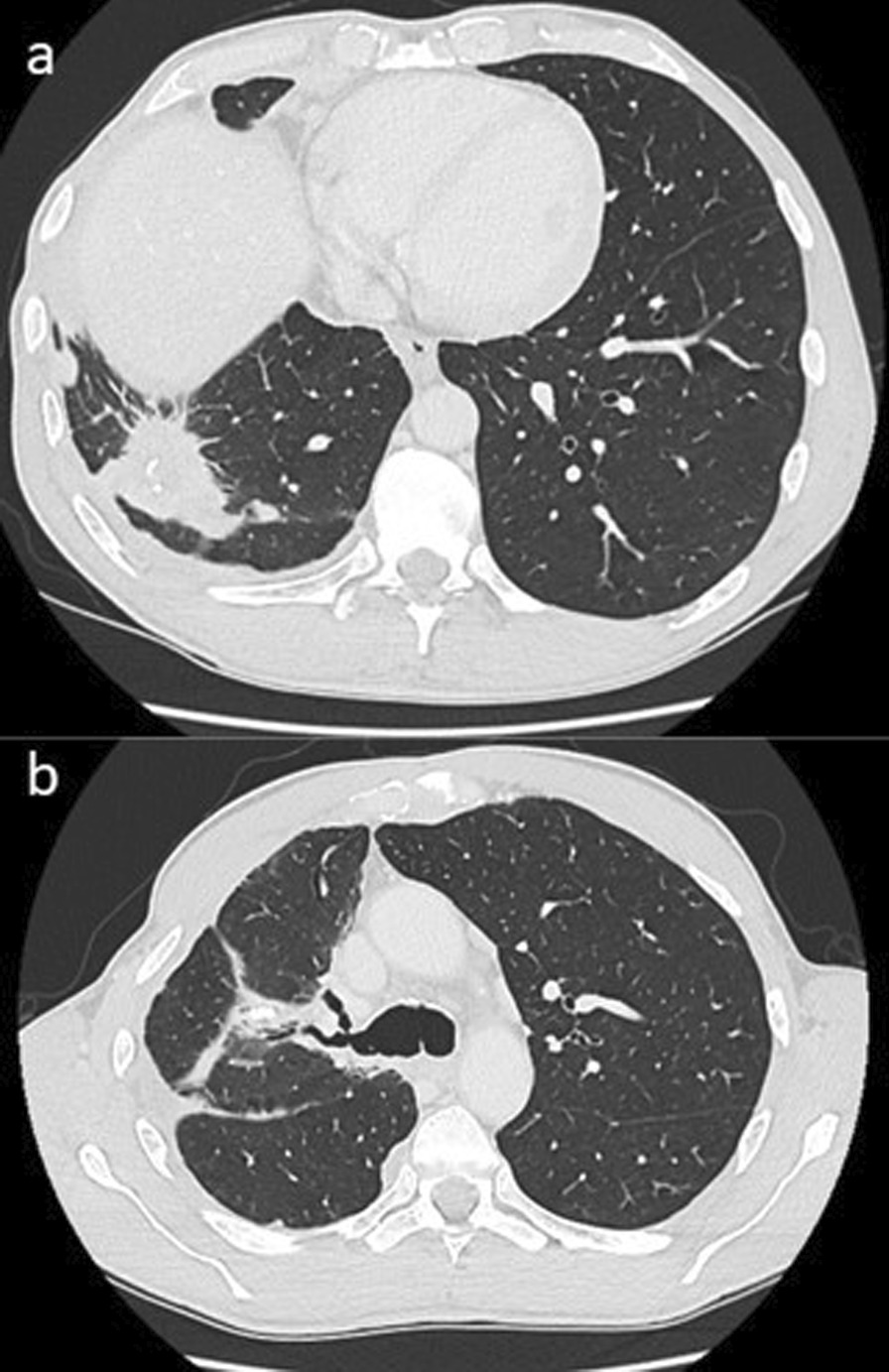
**a** Interval enlargement of the right lower lobe mass. **b** Pulmonary changes in the right upper lobe remained stable

At 18 months post-SABR in October 2021, the CT reported a 10–15% increase in size of the right lower lobe mass (Fig. 7). Other findings were stable. In January 2022, CT and a subsequent PET-CT confirmed the development of diffuse metastatic disease with adrenal, bone and left lung metastases as well as progressive pleural disease. He was admitted under the palliative care unit for pain management and received 20 Gy in 5 fractions for right chest wall pain. On examination he was found to have widespread pain in the back, arms and hips. There was numbness in both upper limbs, the left buttock and the right lateral thigh. He was hypercalcaemic with a corrected calcium of 3.69. Unfortunately, a CT head performed to investigate delirium identified a cerebellar metastasis causing effacement of the 4th ventricle, and the patient died a few days later in February 2022, 5½ years after his initial treatment. An autopsy was not performed.

## Discussion

We present an unusual case of adjuvant lung SABR for three microscopically margin positive (R1) pulmonary metastases from PC, achieving prolonged local control with no side effects. Although he eventually developed widespread recurrence with chest wall pain attributed to pleural and rib metastases, crucially, this patient was able to avoid the added toxicity of palliative systemic therapy. This was of particular concern given his poor tolerance of chemotherapy at initial treatment.

The cause of isolated treatment failure in the right lower lobe region is not clear. We propose the possibility of radioresistant clones, or treatment related factors such as dose limitations due to the proximity of the PTV to the chest wall.

PC rarely leads to isolated lung metastases, the natural history of which is not well described in the literature. The survival in these patients appears to be more favourable than in those with metastases to other solitary organs. The pattern of isolated pulmonary metastases is also more common in females. Other independent predictors for lung metastases in PC include poorly differentiated disease and large primary tumour size. These observations would suggest that the pathogenesis of metastatic PC with lung-only metastases may be distinct from that in other metastatic sites, with a more indolent natural history.

The treatment strategy for oligometastases is controversial and particularly challenging for patients with delayed recurrence after resection of primary PC with curative intent. The indications for metastectomy or SABR for pulmonary oligometastases in PC are not as well defined as for colorectal cancer with isolated pulmonary metastatic disease [3]. However, recent advances in effective therapy for PC have led to the emergence of individual patients where metastectomy or SABR may potentially be indicated.

Surgical resection for isolated late pulmonary metastases in PC has been described in single case reports [4], and small retrospective series [5, 6]. Arnaoutakis *et al.* [6] reported that metastectomy for isolated PC lung metastases is safe and effective. Compared to the non-metastectomy patients, the median overall survival was significantly improved (52 vs. 22 months, *P* = 0.04). Additionally, there was a trend to improved post-relapse survival (18.6 vs*.* 7.5 months).

While the role of lung SABR for medically inoperable oligometastases for more common primary malignancies such as breast, colorectal and renal cell carcinoma has been well addressed in various institutional series [7–10], there are only a few case series and case reports in relation to PC [6, 11]. A retrospective cohort study from Italy, analysed the prognostic impact of resection margin status on long term survival with lung metastectomy for colorectal cancer. Univariate and multivariate analyses showed that a narrow resection margin was an independent prognostic factor for worse survival (*P* = 0.006 and HR 3.4, *P* = 0.009) [12]. However, to the best of our knowledge, no previous study has addressed the impact of margin status for PC, nor has there been a report on adjuvant lung SABR post R1 resection for PC.

SABR enables the delivery of extremely precise radiation, allowing sparing of normal organs more efficiently whilst simultaneously delivering a higher biological dose over a shorter treatment time than conventional radiotherapy, hence improving the therapeutic ratio. Additionally, given the typically poor prognosis of patients like the one discussed herein, SABR is able to minimize time spent undergoing treatment, thereby potentially improving quality of life.

## Conclusion

SABR in this case has demonstrated a safe adjuvant therapeutic option for this patient with isolated pulmonary metastases from PC after microscopically positive metastectomy. The efficacy of this approach, however, remains unproven. It is difficult to conclude that this treatment has been successful as there was evidence of local recurrence at one of the SABR sites as well as subsequent multifocal disease and distant metastases. Systemic therapy will likely continue to form the standard of care in these patients. In patients with contraindications to chemotherapy, we propose that metastasis-directed therapy is a suitable alternative in well-selected patients. We believe that referral to a Multidisciplinary Team clinic is a crucial requirement to consider the potential therapeutic value of SABR for such cases. Further research on lung-only metastases is warranted as this patient cohort has been shown to follow a more favourable natural history than those with multi-organ metastases.

## Data Availability

Not applicable.

## Abbreviations

PC: Pancreatic cancer
SABR: Stereotactic ablative body radiation therapy
CT: Computed tomography
PET: Positron emission tomography
FDG: FluoroDeoxyGlucose
TTF1: Thyroid transcription factor 1
ECOG: Eastern Cooperative Oncology Group
FEV1: Forced expiratory volume in 1 s
4D-CT: 4-Dimensional-CT
PTV: Planning target volume
VMAT: Volumetric modulated arc therapy
